# Extracapsular Nodal Extension as a Key Prognostic Factor in FIGO IIIC Endometrial Carcinoma: A Clinic–Pathologic and Molecular Analysis

**DOI:** 10.3390/cancers18172770

**Published:** 2026-08-26

**Authors:** Lingmei Li, Changyu Geng, Ningrui Feng, Shi Zhang, Lu Cao, Wenfeng Cao, Bo Yang, Yaomei Ma, Su Zhang, Huiting Xiao, Ying Chen, Lei Zhang, Chunxiang Li, Xiaofeng Li, Yang Li, Lisha Qi

**Affiliations:** 1 Department of Pathology, Tianjin Medical University Cancer Institute & Hospital, National Clinical Research Center for Cancer, Tianjin’s Clinical Research Center for Cancer, Key Laboratory of Cancer Prevention and Therapy, Tianjin, Tianjin 300060, China; lilingmei@tjmuch.com (L.L.); gengchangyu@tmu.edu.cn (C.G.); rui03@tmu.edu.cn (N.F.); zhangs03@tmu.edu.cn (S.Z.); caolu@tjmuch.com (L.C.); caowenfeng@tjmuch.com (W.C.); yangbo@tjmuch.com (B.Y.); 2 Department of Gynecology Oncology, Tianjin Medical University Cancer Institute & Hospital, National Clinical Research Center for Cancer, Tianjin’s Clinical Research Center for Cancer, Key Laboratory of Cancer Prevention and Therapy, Tianjin, Tianjin 300060, China; mayaomei@tjmuch.com (Y.M.); zhangsu@tmu.edu.cn (S.Z.); xiaohuiting@tjmuch.com (H.X.); lychenying2004@tmu.edu.cn (Y.C.); zhanglei3805@tjmuch.com (L.Z.); 3 Department of Diagnostic and Therapeutic Ultrasonography, Tianjin Medical University Cancer Institute & Hospital, National Clinical Research Center for Cancer, Tianjin’s Clinical Research Center for Cancer, Key Laboratory of Cancer Prevention and Therapy, Tianjin, Tianjin 300060, China; lichunxiang@tjmuch.com; 4 Department of Molecular Imaging and Nuclear Medicine, Tianjin Medical University Cancer Institute & Hospital, National Clinical Research Center for Cancer, Tianjin’s Clinical Research Center for Cancer, Key Laboratory of Cancer Prevention and Therapy, Tianjin, Tianjin 300060, China; xli03@tmu.edu.cn; 5 Department of Pathology, Hotan District People’s Hospital, Hotan 848000, China; mawenjuan@tmu.edu.cn

**Keywords:** endometrial carcinoma, International Federation of Gynecology and Obstetrics 2023, lymphatic metastasis, extracapsular nodal extension, molecular classification

## Abstract

Background: Assessment of lymph node involvement was incorporated into the 2023 International Federation of Gynecology and Obstetrics (FIGO) staging system as a critical determinant of patient management and prognostication. This study aims to validate the prognostic significance of metastatic lymph node characteristics and their correlation with molecular classification. Methods: We conducted a retrospective analysis of 131 patients with stage IIIC endometrial carcinoma who underwent surgical intervention at the Tianjin Medical University Cancer Institute and Hospital from 2010 to 2025. The patients were stratified based on various characteristics of metastatic lymph nodes. Progression-free survival (PFS) was estimated using Kaplan–Meier curves, and group comparisons were performed using the log-rank test. Univariate Cox proportional hazards regression was conducted to identify independent prognostic factors for PFS. Results: In accordance with the 2023 FIGO staging system, within the stage IIIC cohort, patients with concurrent pelvic and para-aortic lymph node metastases and those in the stage IIIC1 cohort with macrometastasis demonstrated decreased PFS (HR = 2.748; 95% CI = 1.227–6.151; *p* = 0.011, and HR = 4.039; 95% CI = 1.232–13.240; *p* = 0.021, respectively). Furthermore, patients with lymph nodes showing extracapsular extension had significantly reduced PFS in the stage IIIC, stage IIIC1, and stage IIIC1ii cohorts (HR = 10.12; 95% CI = 2.38–42.99; *p* < 0.001; HR = 3.58; 95% CI = 1.242–10.31; *p* = 0.011; and HR = 2.32; 95% CI = 0.83–6.71; *p* = 0.037, respectively). The number of involved lymph nodes, the largest diameter of metastatic foci in lymph nodes, and the presence or absence of extracapsular extension of metastatic lymph nodes were significantly different among the NSMP, MMRd, and p53abn molecular classifications (*p* = 0.033, *p* < 0.001, and *p* = 0.002, respectively). Patients with extracapsular extension of metastatic lymph nodes had worse PFS than those without in the MMRd group (*p* = 0.018). Conclusions: Extracapsular extension (ECE) of metastatic lymph nodes is the strongest predictor of worse progression-free survival in stage IIIC endometrial carcinoma, especially in the IIIC1 and MMRd subgroups, supporting its value for risk stratification and prognostic assessment.

## Simple Summary

Per the FIGO 2023 staging system, stage IIIC endometrial carcinoma is subdivided into IIIC1 and IIIC2 based on the site of nodal involvement (pelvic vs. para-aortic), and further classified into i and ii according to the size of intra-nodal metastatic deposits. We evaluated nodal characteristics and molecular classification in relation to clinical outcomes. The most consistent and clinically significant finding was that extracapsular extension (ECE) of metastatic lymph nodes was associated with worse progression-free survival (PFS), both in the overall stage IIIC cohort (HR = 13.25, 95% CI = 2.36–74.25, *p* < 0.001) and in the IIIC1 subgroup (HR = 3.58; 95% CI = 1.242–10.309; *p* = 0.011). ECE is defined as tumor growth that extends beyond the lymph node capsule into the perinodal adipose or fibrous tissue. The ECE of metastatic lymph nodes was associated with worse progression-free survival (PFS) in the mismatch-repair-deficient (MMRd) subgroup (*p* = 0.018).

## 1. Introduction

Endometrial carcinoma (EC) represents one of the most prevalent malignancies affecting women globally, with an increasing global incidence rate [1,2]. Currently, surgical intervention is the primary treatment modality for EC, with decisions regarding adjunctive therapy guided by pathological classification and staging [3,4]. Lymph node metastases (LNMs) are the predominant metastatic pattern in EC and serve as an independent prognostic factor [5]. Approximately 20% of patients exhibit LNMs, and the 5-year disease-specific survival rate is highly heterogeneous, ranging from 18% to 71% [6,7]. Therefore, thorough risk stratification is essential for these patients, as it significantly influences their response to adjuvant treatment and survival.

The revised 2023 International Federation of Gynecology and Obstetrics (FIGO) staging system has classified pelvic lymph node metastasis (LNM) and para-aortic lymph node metastases into stages III C1 and III C2, while further refining subgroups by designating “i” for micrometastasis and “ii” for macrometastasis according to the dimensions of intra-lymph node metastasis [8]. Some studies have demonstrated that the 2023 staging system, incorporating refined parameters for LNM, provides superior stratification of patient outcomes compared with the 2009 staging system [9,10]. In addition to the anatomical positioning of metastatic lymph nodes and the dimension of intra-lymph node metastases, several studies have investigated the prognostic significance of the number of metastatic lymph nodes [11,12,13]. However, these studies are limited in scope, with some findings being inconsistent or even contradictory. In a survey developed by the International Society of Gynecologic Pathologists, 31% of pathologists and 41% of clinicians agree that the extracapsular extension of the lymph node should be sub-staged [14]. However, the prognostic significance of extracapsular lymph node extension in EC remains unclear.

Given the significant advances that have been made in the understanding of the molecular underpinnings of EC in the last decade, the 2023 FIGO committee advocates the performance of molecular classification (*POLE*-mutated, mismatch repair deficient, non-specific molecular profile, p53 abnormal) in all EC cases, not only for prognostic risk stratification but also to guide treatment decisions [8]. The 2025 ESGO-ESTRO-ESP Guidelines have established a molecular subtype-driven, full-cycle diagnosis and treatment framework based on the 2023 FIGO new staging system and the latest molecular evidence, thereby providing a globally recognized, evidence-based standard for risk stratification and treatment decision-making in patients with advanced endometrial carcinoma [15]. Nevertheless, research exploring the correlation between the extracapsular extension of LNM and the molecular classification of EC remains limited. The present study aimed to analyze the prognostic significance of extracapsular extension of LNM in the subgroup of EC patients with LNM.

## 2. Materials and Methods

### 2.1. Study Cohort

This is a retrospective study approved by the Ethics Committees of Tianjin Medical University Cancer Institute & Hospital (bc20261004). Patients diagnosed with endometrial carcinoma (EC) who underwent hysterectomy with systematic lymphadenectomy at Tianjin Medical University Cancer Institute & Hospital between June 2010 and June 2025 were included. Exclusion criteria were as follows: patients without lymph node metastasis (LNM) or with inadequate lymphadenectomy for pathological evaluation; those who received preoperative therapy; patients diagnosed with FIGO stage IV disease; cases with only isolated tumor cells (ITCs) in lymph nodes; and individuals with other concurrent malignancies. Consequently, 131 patients were included. The flow diagram for this retrospective study is presented in Supplementary Figure S1.

### 2.2. Evaluation of Pathological Parameters

A retrospective review of the patients’ clinical records and pathological materials was conducted. The collected data included age at diagnosis, tumor dimensions, histological subtype, extent of lymphovascular space invasion, type of adjuvant therapy administered, extent of cervical stroma involvement, adnexal involvement, uterine serosal involvement, recurrence status, and survival duration. The histological subtype and FIGO grade were assessed according to the World Health Organization (WHO) criteria [16]. All histological slides were independently reviewed by two experienced gynecopathologists (Bo Yang and Wenfeng Cao), and the pathological review was standardized. The presence of extracapsular nodal extension (ECE), localization, size, number of metastatic lymph nodes, and the diameter of the metastatic foci within the lymph node were recorded. ECE is defined as tumor growth that extends beyond the lymph node capsule and into the perinodal adipose or fibrous tissue. The histological workflow was clearly defined. Three consecutive sections containing the maximal cross-section of each lymph node were examined, and cytokeratin immunohistochemistry (IHC) was performed concurrently for confirmation. Extracapsular extension (ECE) was defined as the presence of pan-cytokeratin (CK-pan)-positive neoplastic cells extending through the lymph node capsule into the surrounding perinodal adipose or fibrous tissue. The representative histopathological images are presented in Supplementary Figure S2. Only lymph nodes that were excised without division were assessed for size. Adequate pelvic lymphadenectomy and para-aortic lymphadenectomy were defined as the removal of no fewer than 15 pelvic lymph nodes and five para-aortic lymph nodes, respectively. The diameter of the metastatic foci within the lymph node was referenced to the exact definition of micrometastasis vs. macrometastasis (2 mm cutoff for intra-nodal focus size, per FIGO/ISGyP consensus) [8].

### 2.3. Molecular Classification and Immunohistochemistry (IHC)

The molecular classification was performed following the surrogate marker-based diagnostic algorithm of the 5TH WHO, resulting in POLE mutation, mismatch repair deficient (MMRd), p53 abnormal (p53abn), or non-specific molecular profile (NSMP). The methodology for molecular classification is clearly described in Supplementary Figure S3. The formalin-fixed, paraffin-embedded (FFPE) tumor blocks from all cases were assembled and DNA-extracted using the Qiagen FFPE tissue kit. The POLE exonuclease domain (exons 9, 11, 13, and 14) was sequenced to classify POLE mutation cases. They were classified as MMRd and p53abn instances based on the immunohistochemistry (IHC) for MMR proteins and p53. A pathologist selected a representative formalin-fixed paraffin-embedded (FFPE) tumor block for each case. IHC was performed using an automatic immunostaining instrument (Ventana Benchmark XT; Ventana Medical Systems, Tucson, AZ, USA) according to the manufacturer’s instructions. IHC for MMR proteins (MLH1, MSH2, MSH6, PMS2) and p53 was performed using the following antibodies: MutL homolog 1 (MLH1, 1:50, M1), MutS homolog 2 (MSH2, 1:200, G219-1129), MutS homolog 6 (MSH6, 1:100, sP93), PMS1 homolog 2 (PMS2, diluted 1:40, A16-4) and p53 (clone DO-7; 1:300). For p53 IHC, cases were categorized into one of three groups. Wild-type p53 expression was defined as nuclear staining of variable intensity in 1–80% of the tumor. Null expression was characterized by the absence of p53 nuclear staining with a positive internal control. At the same time, overexpression was identified by uniform and intense nuclear staining in at least 80% of tumor cell nuclei. The staining was first validated using positive control nuclei. MMR protein expression in tumor cell nuclei was then evaluated, with emphasis on identifying conserved or lost expression of MLH1, MSH2, MSH6, and PMS2. Tumor cells showed a complete absence of nuclear staining with a positive non-neoplastic internal control. The absence of expression of any of these MMR proteins is referred to as MMR-deficient (MMRd), while the presence of all four MMR proteins is considered MMR-proficient (MMRp). MMR-proficient tumor cells exhibited nuclear positivity. MMR-uncertain cases were tested with real-time PCR. The microsatellite (MSI) markers BAT25, D2S123, BAT26, D17S250, and D5S346, recommended by the National Cancer Institute for MSI detection, were investigated. The fragments were analyzed on an ABI PRISM 3500 genetic analyzer (Applied Biosystems^®^, Foster City, CA, USA). Tumors with MSI in three or more of the five markers are classified as high MSI; those with MSI in fewer than three of the five markers are low MSI; and those with no detectable MSI are microsatellite-stable (Supplementary Figure S4). dMMR status was compared to MMR IHC. MLH1 promoter DNA methylation was analyzed using an EZ DNA methylation-gold kit (Zymo Research, Irvine, CA, USA; Proteigene, Saint-Marcel, France) and PyroMark Q24 CpG MLH1^®^ Kit (Qiagen, Hilden, Germany) in accordance with the manufacturer’s instructions.

### 2.4. Statistical Analysis

Statistical analysis was performed using SPSS Base for Windows (v.15.0, SPSS, Inc., Chicago, IL, USA). Progression-free survival (PFS) and overall survival (OS) were predefined as key endpoints in our study. However, only one death event occurred among the study cohort during the follow-up period, resulting in insufficient statistical power to conduct meaningful analyses for OS. Follow-up was censored on 1 January 2026. For patients who experienced disease progression, the date of progression was used as the endpoint. Disease progression was determined by reviewing clinical medical records and telephone follow-up data. Therefore, the subsequent statistical analyses focused primarily on PFS, which had sufficient events to support robust conclusions. Survival outcomes, including PFS, were analyzed using the Kaplan–Meier method with the log-rank test for group comparisons. The proportional hazards assumption was tested in our study cohort and found to be satisfied. To identify independent prognostic factors for PFS, we performed univariate Cox proportional hazards regression. Variables with *p*-values < 0.05 in the univariate analysis were subsequently included in a multivariate Cox proportional hazards regression model to estimate hazard ratios (HRs) with 95% confidence intervals (CIs). A *p*-value less than 0.05 was considered statistically significant. Power calculation was performed based on the observed hazard ratio, actual sample size, and a two-sided significance level of α = 0.05 (Supplementary Tables S1 and S2).

## 3. Results

### 3.1. Baseline Characteristics

A total of 131 patients with stage IIIC endometrial carcinoma (EC) were included in this study. The patient characteristics are presented in Supplementary Table S3. The median follow-up duration was 32.56 months, with the longest at 118.47 months. Among the 131 patients with lymph node metastasis (LNM) included in this study, 26 (19.8%) experienced disease recurrence or progression. Additionally, all patients received adjuvant chemotherapy, while 86 (65.6%) underwent concurrent chemoradiotherapy (45.8%), targeted therapy (8.4%), or immunotherapy (11.5%).

Among the 2901 resected lymph nodes from 131 patients, 336 were positive, including 215 pelvic and 121 para-aortic lymph nodes. The median number of resected lymph nodes per patient was 22. Given that no significant difference in PFS was observed between chemotherapy with and without other adjuvant therapy in the study cohort (*p* = 0.678; Supplementary Figure S5), the survival significance of metastatic lymph node characteristics can be evaluated more rigorously.

### 3.2. The Extracapsular Extension of Metastatic Lymph Nodes Is Associated with a Poor Prognosis

**Figure 1:**
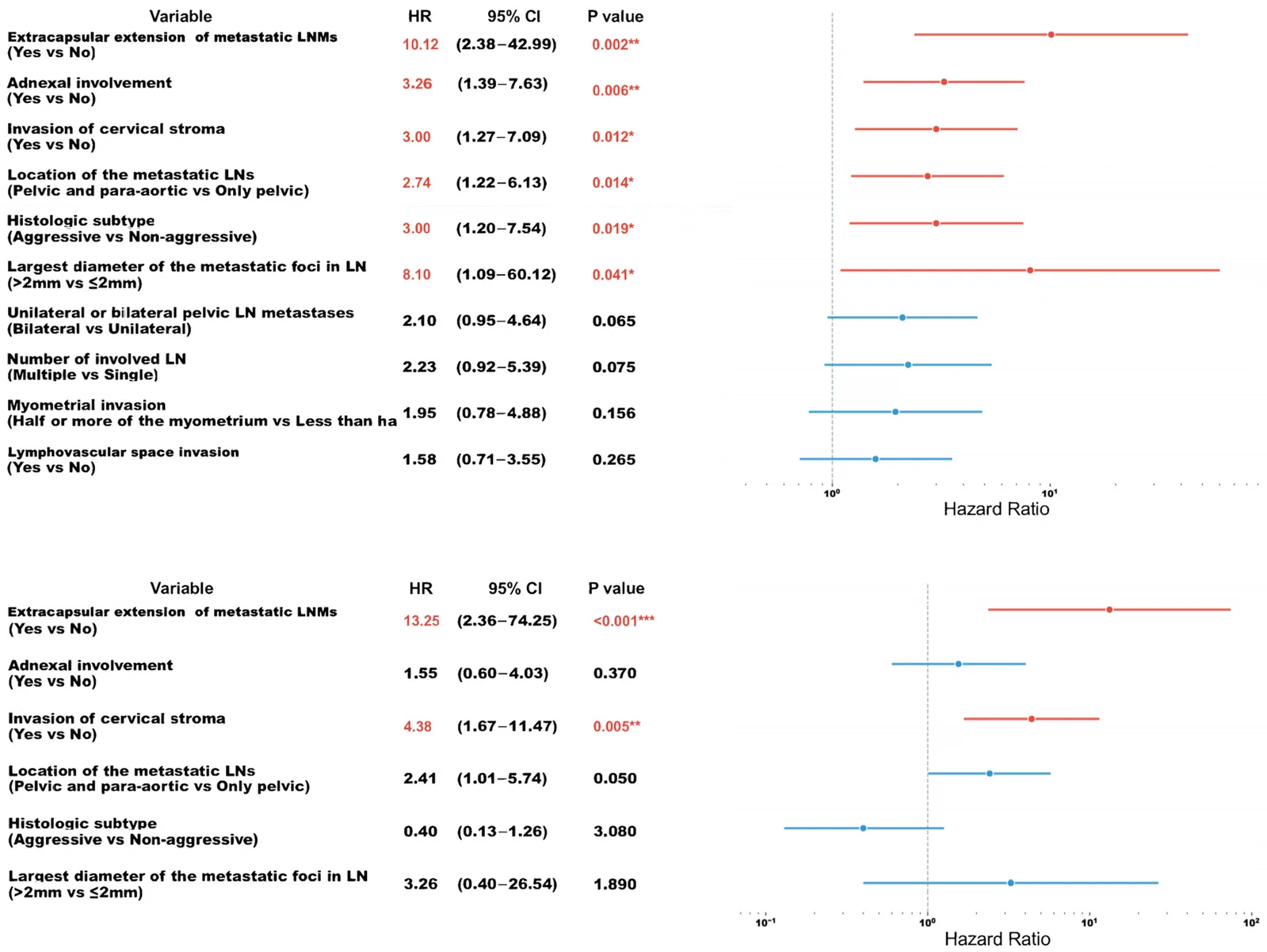
The univariate (up) and multivariate (down) Cox regression analyses for PFS in stage IIIC cohort endometrial carcinoma patients. * *p* < 0.05, ** *p* < 0.01, *** *p* < 0.001. Internal validation was carried out using bootstrap Cox proportional hazards modeling with 1000 bootstrap samples. HR, hazard ratio; 95% CI, 95% confidence interval of the estimated HR intervals.

The univariate and multivariate Cox regression analyses exploring the factors influencing patients’ PFS are meticulously presented in Figure 1. Moreover, univariate Cox regression analysis indicated that extracapsular extension of metastatic LNs, adnexal involvement, invasion of cervical stroma, location of metastatic LNs, histologic subtype, and the largest diameter of metastatic foci in the LNs were all associated with poorer PFS. More importantly, multivariate Cox regression models strongly verified that extracapsular extension of the metastatic LNs (HR = 13.25, 95% CI = 2.36–74.25, *p* < 0.001) and invasion of cervical stroma (HR = 4.38,95% CI = 1.67–11.47, *p* = 0.005) were independently associated with worse PFS. This finding indicates that extracapsular extension of metastatic LNs and invasion of the cervical stroma are associated with a higher risk of poor patient outcomes.

### 3.3. Impact of Characteristics of Metastatic Lymph Nodes on the PFS of Stage IIIC Endometrial Carcinoma Patients

The characteristics of lymph nodes are presented in Table 1. A total of 94 (71.8%) patients exhibited unilateral pelvic lymph node involvement, while 37 (28.2%) patients demonstrated bilateral pelvic lymph node involvement. No statistically significant difference in PFS was observed between the two groups (HR = 2.106; 95% CI = 0.955–4.644; *p* = 0.059; Supplementary Figure S6A). There were 51 (41.2%) patients with single lymph node involvement and 77 (58.8%) with multiple lymph node involvement; however, no significant difference in PFS was observed between the two groups (HR = 2.23; 95% CI = 0.92–5.39; *p* = 0.068; Supplementary Figure S6B). In the stage IIIC and stage IIIC1 cohorts, the survival analysis showed that patients with cervical stromal invasion had poorer PFS (HR = 3; 95% CI = 1.27–7.09; *p* = 0.008; HR = 3.01; 95% CI = 1.00–9.09; *p* = 0.037, respectively). In the stage IIIC2 and stage III1ii subgroups, no significant difference was observed between patients with cervical stroma invasion and those without (*p* = 0.163 and *p* = 0.070, respectively; Supplementary Figure S7A–D).

**Figure 2:**
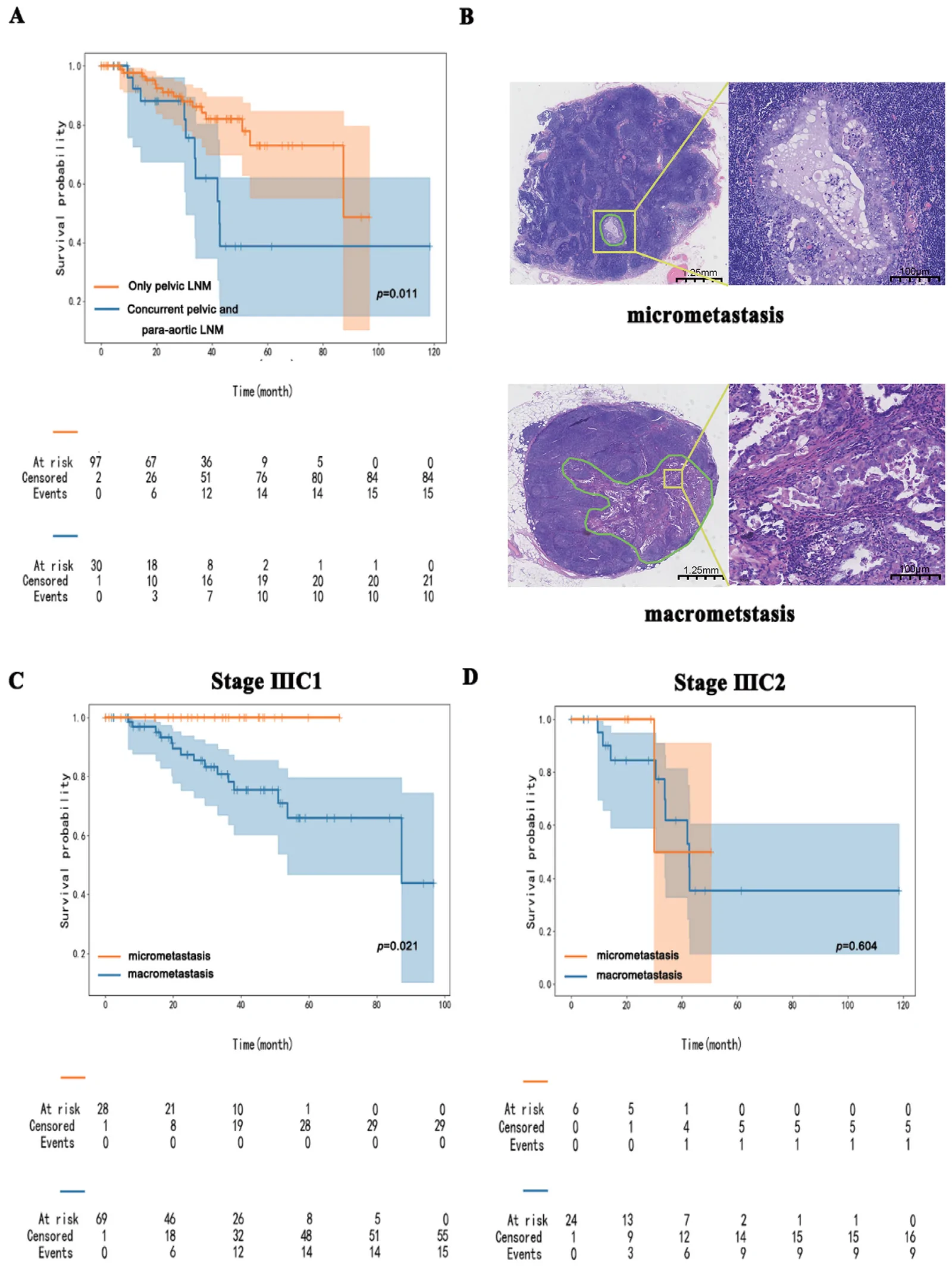
Prognostic significance of the location of the metastatic lymph nodes and the largest diameter of the metastatic foci in lymph nodes. (**A**) Kaplan–Meier curves estimating PFS by metastatic lymph node location in the stage IIIC cohort. (**B**) Representative histopathological images of micrometastasis and macrometastasis in lymph nodes (marked with a green line). The yellow boxes and the magnified image on the right illustrate metastatic cancer. (**C**) Kaplan–Meier curves estimating PFS by lymph node micrometastasis or macrometastasis in the stage IIIC1 cohort. (**D**) Kaplan–Meier curves estimating PFS by lymph node micrometastasis or macrometastasis in the stage IIIC1 cohort. LNM, lymph node metastasis.

As expected, the PFS in the 31 (23.7%) patients with metastasis concurrently involving pelvic and para-aortic lymph nodes was significantly poorer compared to the 100 (76.3%) patients with metastasis confined to the pelvic lymph nodes (HR = 2.74; 95% CI = 1.22–6.13; *p* = 0.011, Figure 2A). In subgroup analyses, it was observed that for patients with metastasis confined to the pelvic lymph node, PFS was superior in those (29/100, 29.0%) with micrometastasis (0.2–2 mm in size and/or more than 200 cells) compared to those (71/100, 71.0%) with macrometastasis (larger than 2 mm) (HR = 34.48; 95% CI = 0.228; *p* = 0.021, Figure 2B,C). Among patients with para-aortic LNM, 6 of 31 (19.4%) had micrometastasis, and 25 (78.6%) had macrometastasis. While the PFS of micrometastasis was superior to that of macrometastasis, the difference did not reach statistical significance. (HR = 0.058; 95% CI = 0.072–0.488; *p* = 0.604, Figure 2D). The disparity in case numbers between the two groups may have precluded detection of a statistically significant difference in survival.

**Figure 3:**
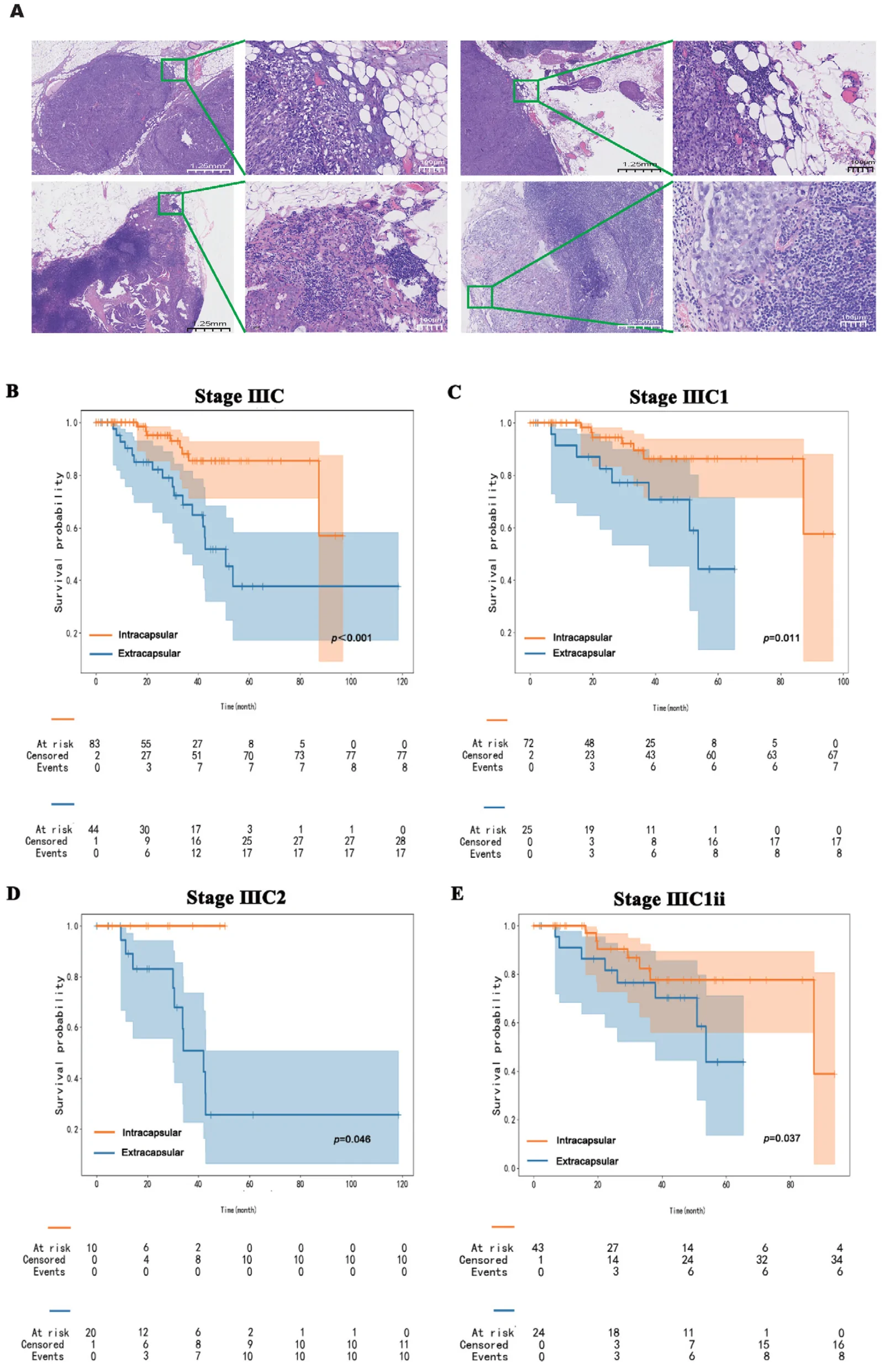
Prognostic significance of the extracapsular extension of lymph nodes. (**A**) Representative histopathological images of extracapsular extension of lymph nodes. The green boxes and the magnified image on the right illustrate metastatic cancer (enlarged display in green box). (**B**) Kaplan–Meier curves estimating PFS by extracapsular versus intracapsular lymph node extension in the stage IIIC cohort. (**C**) Kaplan–Meier curves estimating PFS by extracapsular versus intracapsular lymph node extension in the stage IIIC1 cohort. (**D**) Kaplan–Meier curves estimating PFS by extracapsular versus intracapsular lymph node extension in the stage IIIC2 cohort. (**E**) Kaplan–Meier curves estimating PFS by extracapsular versus intracapsular lymph node extension in the stage IIIC1ii cohort.

Among the 131 patients with endometrial carcinoma, 45 (34.4%) exhibited extracapsular extension of the lymph node (Figure 3A), while 86 (65.6%) did not. The clinicopathological characteristics of extracapsular nodal extension in endometrial carcinoma patients were shown in Supplementary Table S4. There was a significant difference in PFS between the two groups (HR = 10.12; 95% CI = 2.38–42.99; *p* < 0.001; Figure 3B). To further elucidate the prognostic implications of extracapsular extension in affected lymph nodes, we conducted subgroup analyses. It was observed that in patients with stage IIIC1 and IIIC2, those with extracapsular lymph node extension had inferior PFS compared with those without (HR = 3.57; 95% CI = 1.24–10.53; *p* = 0.011 and HR = 4.255; 95% CI = 0.538–33.673; *p* = 0.046, respectively; Figure 3C,D). The same is true in the further subdivided IIIC1ii groups (HR = 4.31; 95% CI = 0.15–1.25; *p* = 0.037, Figure 3E). In stage IIIC2i and IIIC2ii patients, those with extracapsular extension of the lymph node had a lower PFS than those without. However, the difference is not statistically significant (HR = 2.352; 95% CI = 0.498–11.180; *p* = 0.317, and HR = 4.21; 95% CI = 0.05–3.04; *p* = 0.403, Supplementary Figure S8). In the 3IIIC1i subgroup, only one case showed disease progression, precluding statistical analysis.

### 3.4. The Characteristics of Metastatic Lymph Nodes Among Different Molecular Classifications

**Figure 4:**
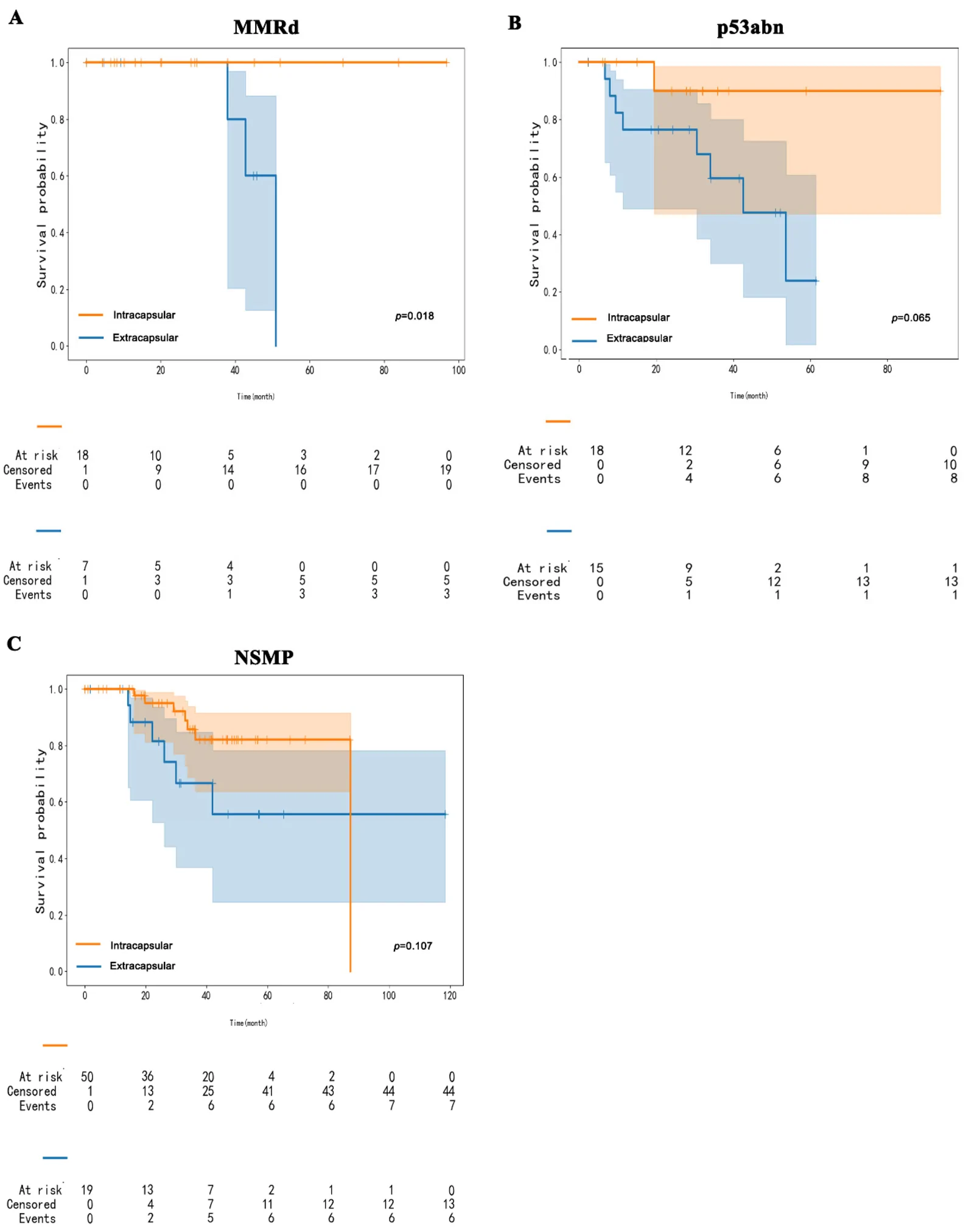
Prognostic significance of the extracapsular extension of lymph nodes in molecular classification. (**A**) Kaplan–Meier curves estimating PFS by extracapsular versus intracapsular lymph node extension in patients with the MMRd molecular subtype. (**B**) Kaplan–Meier curves estimating PFS by extracapsular versus intracapslar lymph node extension in patients with the p53abn molecular subtype. (**C**) Kaplan–Meier curves estimating PFS by extracapsular/intracapsular lymph node extension in patients with NSMP molecular subtype. MMRd: mismatch repair-deficient; p53abn: p53 abnormal; NSMP: non-specific molecular profile.

Among the 131 EC patients, none had molecular features of a POLE mutation. The distribution of the remaining molecular classifications was as follows: mismatch repair deficient (MMRd) in 28 cases, p53 abnormal (p53abn) in 33 cases, and non-specific molecular profile (NSMP) in 70 cases. The characteristics of metastatic lymph nodes in cohorts with MMRd, p53abn, and NSMP molecular classification are presented in Table 2. The number of involved LNs, the extracapsular extension of LN, and the largest diameter of the metastatic foci in LN were significantly different among NSMP, MMRd, and p53abn molecular classifications (*p* = 0.033, *p* = 0.002, and *p* < 0.001, respectively). Further analysis revealed that in the MMRd molecular subgroup, patients exhibiting extracapsular extension of lymph node demonstrated significantly poorer PFS compared to those without such invasion (*p* = 0.018, Figure 4A). Within the p53abn subgroup, a similar trend was observed; however, the difference did not achieve statistical significance (HR = 5.747; 95% CI = 0.714–45.46; *p* = 0.065, Figure 4B), potentially due to the limited number of cases exhibiting tumor progression within the p53abn subtype. In the NSMP molecular subgroups, no significant difference was observed between patients with or without extracapsular extension of lymph nodes (HR = 2.44; 95% CI = 0.81–7.35; *p* = 0.107, Figure 4C).

## 4. Discussion

In this study, we comprehensively investigate the prognostic significance of metastatic lymph node characteristics in patients with surgically treated endometrial carcinoma (EC). The results of this study confirmed the prognostic importance of micro- and macro-metastasis in pelvic and para-aortic lymph node metastases, as refined by the 2023 FIGO staging system. Of note, the extracapsular extension of metastatic lymph nodes was associated with decreased PFS in EC patients with stage IIIC disease. In addition, we further analyzed the correlation of extracapsular extension of the metastatic lymph node and the molecular classification of EC. No patients harboring *POLE* mutations were detected in this cohort. The extracapsular extension of metastatic lymph nodes showed significant differences across the three molecular classifications. In the mismatch repair-deficient (MMRd) classification, patients with extracapsular extension of metastatic lymph nodes had poorer PFS than those without it (Supplementary Table S5).

Compared with the 2009 version, the 2023 FIGO staging system revised the stage for patients with lymph node metastasis (stage IIIC) based on the site and size of the involved lymph node. Several studies have investigated this new FIGO 2023 staging system for EC. Mayumi et al. investigated 265 patients with EC. A subgroup analysis of stage IIIC patients revealed a significant difference in 5-year OS between patients with pelvic and those with para-aortic lymph node metastasis. Nevertheless, no significant difference was detected between micrometastasis and macrometastasis [17].Varol performed a study in 120 EC cases of stage IIIC and concluded that patients with only pelvic lymph node metastasis had higher 5-year DFS and rates than those with para-aortic nodal metastasis [12]. Matsuo et al. conducted a study of 5473 patients with advanced EC in the National Cancer Institute’s Surveillance, Epidemiology, and End Results (SEER) [10]. They concluded that patients with macro-metastasis in lymph nodes had a higher EC-specific mortality than those with micro-metastasis. Another study by Virginia et al. evaluated the oncological outcomes of 270 patients with endometrial carcinoma. It showed that patients with macrometastasis had significantly worse PFS and 5-year OS than those with low-volume lymph node disease [18]. Consistent with previous reports, our findings emphasize the significance of micrometastasis versus macrometastasis in the regional lymph nodes, as refined in the FIGO 2023 classification. Recent studies further verified the inclusion of lymph node parameters in the FIGO classification and confirmed that the site of lymph node metastasis correlates with poorer prognosis [19,20].

The extracapsular extension of a metastatic lymph node indicates the high invasiveness of tumor cells. It has been reported to have a high prognostic value in patients with esophageal cancer, vulval squamous cell carcinoma, papillary thyroid carcinoma, etc. [21,22,23]. However, only three studies have tackled this issue in EC. Makio evaluated the clinicopathologic prognostic factors for EC with LNM in a cohort of 24 patients [24]. Among these, 12 patients exhibited tumor invasion into the perinodal fat, which was associated with significantly poorer survival outcomes compared to cases without such invasion. Giorgia et al. conducted a study of 103 patients who underwent primary surgery with sentinel lymph node mapping and had at least one positive node [25]. They reported that the extracapsular location of the sentinel lymph nodes was associated with an increased risk of multiple positive sentinel lymph node metastases. In a study of 96 EC cases, Valentina and colleagues reported that patients with extracapsular metastasis were more likely to have positive para-aortic nodes than those with intracapsular metastasis [26]. In the current study, we found that ECE of the metastatic lymph node was associated with a significant decrease in PFS in EC patients with stage IIIC disease. Mechanistically, ECE represents an aggressive tumor biological phenotype characterized by disruption of the lymph node capsule integrity. This disruption facilitates tumor invasion into adjacent adipose and vascular tissues, promotes micrometastasis, and ultimately increases the risk of recurrence. These processes are consistent with established evidence that capsular breach enables direct access to perinodal lymphatic and blood vessels, thereby accelerating systemic dissemination. Together, they provide a plausible explanation for the poorer survival outcomes observed in patients with ECE, even when tumor stage and lymph node metastatic burden are comparable. Clinically, routine pathological reporting of extracapsular extension (ECE) can refine the existing pathological evaluation and risk stratification systems for endometrial carcinoma (EC), overcoming the limitations of current FIGO staging and ESGO/ESTRO/ESP risk criteria, which fail to distinguish heterogeneous prognostic risks among lymph node-positive patients. Such reporting enables individualized postoperative management, allowing intensified adjuvant therapy and stringent surveillance for high-risk patients with ECE while avoiding overtreatment for those without ECE.

Regarding the number and location of pelvic lymph nodes in patients with endometrial carcinoma, the current study found no prognostic significance for these features for PFS. There are conflicting results. Several studies have concluded that the number of involved lymph nodes is a prognostic and predictive factor for EC patients. However, the cut-off values for positive lymph nodes in these studies were not consistent [26,27]. Differences in the number of cases and differences in patients’ backgrounds may have influenced the results.

Molecular classification has revolutionized our understanding of EC and has been integrated into the FIGO stage I and stage II staging systems to improve prognosis evaluation and guide treatment decisions [28]. Of note, molecular classification is required for proper risk group allocation, and several studies have indicated that it may influence the prognosis of advanced EC. Mayumi et al. reported that in stage IIIC EC (2008 FIGO), the 5-year OS of patients with p53 abnormalities was significantly worse than that of patients with the p53 wild-type pattern [17]. Another study revealed that patients with metastatic lymph nodes showing aberrant p53 expression had lower disease-free survival than those with wild-type p53 expression [29]. In our cohort, stage IIIC EC patients with p53 mutations did not have poorer PFS than those with p53 wild-type, and there was no significant difference in PFS among patients in the NSMP, MMRd, and p53abn groups. Notably, none of the 131 EC patients with lymph node metastasis harbored a POLE mutation. This observation is consistent with the known favorable prognosis of POLE-mutant endometrial carcinoma, which typically presents with less aggressive features and a lower likelihood of lymph node involvement. Indeed, prior studies have reported that POLE-mutant EC constitutes an extremely low proportion (1–3%) of cases and is associated with a more favorable prognosis. Our results are in line with these findings [30,31].

To our knowledge, no studies have analyzed the relationship between diverse metastatic lymph node features and the molecular typing of EC. In this study, the presence or absence of extracapsular extension of metastatic lymph nodes differed significantly among the NSMP, MMRd, and p53abn molecular classifications. Further analysis showed that, in the MMRd group, patients with extracapsular extension of metastatic lymph nodes had a worse PFS than those without [32]. Our data extend this framework by demonstrating that extracapsular extension further stratifies the MMRd subgroup into a high-risk subset with inferior PFS. This finding supports the guidelines’ advocacy for a multifactorial risk assessment that integrates molecular subtype and pathological features into treatment decision-making in stage III disease.

We investigated the prognostic significance of multiple characteristics of metastatic lymph nodes in patients with stage IIIC disease according to the new 2023 FIGO staging system. Compared to the previous studies, which predominantly focused on the prognostic implications of individual lymph node features, this study encompassed a broader range of features, including the site and size of metastatic lymph nodes as defined by the updated 2023 FIGO staging system, as well as the number of metastatic lymph nodes, unilateral/bilateral pelvic lymph node involvement, and extracapsular extension of metastatic lymph nodes. Studies investigating extracapsular nodal extension (ECE) remain relatively limited in gynecologic neoplasms. Matteo et al. conducted a study in 111 ovarian cancer cases and reported that isolated ECE was associated with a potentially less favorable prognosis [33]. In contrast, our study evaluates the prognostic significance of ECE in stage IIIC endometrial carcinoma according to the 2023 FIGO staging system. Also, this study is the first to analyze the correlation between metastatic lymph node characteristics and the molecular classification of EC.

This study has several limitations. First, it was a single-center retrospective analysis. Second, patients with POLE exonuclease domain mutations were excluded; thus, the prognostic significance of lymph node features could not be assessed in this subgroup, which had a favorable prognosis and low rates of lymph node metastasis. Third, progression-free survival (PFS) was used as the primary endpoint, whereas overall survival (OS) data were immature due to insufficient follow-up, limiting comprehensive prognostic evaluation. Fourth, the relatively high hazard ratio and wide confidence intervals observed for extracapsular extension in the multivariate analyses were likely attributable to the limited number of progression events. Although the results were statistically significant, the estimated effect size should be interpreted with caution. Fifth, subgroup analyses stratified by molecular subtypes (MMRd, p53abn, and NSMP) demonstrated promising clinical implications; however, these analyses were based on small subsets and should therefore be considered exploratory, warranting further validation in large multicenter cohorts. Sixth, several non-significant results yielded *p*-values approaching 0.05. Although these differences did not reach statistical significance, they reflected consistent biological trends in PFS that were likely attenuated by the cohort’s limited sample size. Finally, the study cohort spanned from 2010 to 2025, a period during which targeted therapies and immunotherapies evolved rapidly [34,35].The relatively small number of patients who received modern adjuvant regimens may have limited the statistical power to detect their impact on PFS. Although the homogeneous study population and standardized pathological assessment strengthened our results, further large-scale prospective studies are needed to validate these findings.

## 5. Conclusions

In conclusion, this study comprehensively elucidates the robust and independent prognostic value of lymph node extracapsular extension (ECE) in endometrial carcinoma (EC) within the 2023 FIGO staging system. Beyond the metastatic lymph node site and intranodal metastasis size adopted by the current FIGO staging criteria, ECE serves as a critical supplementary prognostic biomarker for EC. Notably, stage IIIC EC patients with mismatch repair deficiency (MMRd) exhibit significantly worse prognosis when presenting ECE. Clinically, routine pathological reporting of extracapsular extension (ECE) can refine the existing pathological evaluation and risk stratification systems for endometrial carcinoma (EC), overcoming the limitations of current FIGO staging and ESGO/ESTRO/ESP risk criteria, which fail to distinguish heterogeneous prognostic risks among lymph node-positive patients. Such reporting enables individualized postoperative management, allowing intensified adjuvant therapy and stringent surveillance for high-risk patients with ECE while avoiding overtreatment for those without ECE. Collectively, ECE is a powerful, clinically valuable prognostic indicator with great potential to be incorporated into updated staging and risk stratification frameworks, thereby improving the accuracy of prognostic prediction and guiding precise clinical decision-making for EC patients. However, further prospective multicenter studies are warranted to validate externally.

## Figures and Tables

**Table 1:** Characteristics of the metastatic lymph nodes.

Characteristic	N of Patients (%)
Location of the metastatic LNs	
Only pelvic LN metastasis	100 (76.3%)
Concurrent pelvic and para-aortic LN metastasis	31 (23.7%)
Unilateral or bilateral pelvic LN metastasis	
Unilateral	94 (71.8%)
Bilateral	37 (28.2%)
Number of involved LNs	
Single	54 (41.2%)
Multiple	77 (58.8%)
Extracapsular tumor extension of LN	
Absence	86 (65.6%)
Presence	45 (34.4%)
Largest diameter of the metastatic foci in LN	
≤2 mm	35 (26.7%)
>2 mm	96 (73.3%)

LN: Lymph node.

**Table 2:** The characteristics of metastatic lymph nodes in MMRd, p53abn, and NSMP molecular classification. NSMP: non-specific molecular profile; p53abn: p53 abnormal; MMRd: mismatch repair-deficient.

Characteristics	MMRd (N = 28)	p53abn (N = 33)	NSMP (N = 70)	*p* Value
Location of the metastatic LN				0.238
Only pelvic LN metastases	20 (71.4%)	23 (69.7%)	57 (81.4%)	
Concurrent pelvic and para-aortic LN metastases	8 (28.6%)	10 (30.3%)	13 (18.6%)	
Unilateral or bilateral pelvic LN metastases				0.124
Unilateral	23 (82.1%)	20 (60.6%)	51 (72.9%)	
Bilateral	5 (17.9%)	13 (39.4%)	19 (27.1%)	
Number of involved LN				**0.033**
Single	13 (46.4%)	8 (24.2%)	33 (47.1%)	
Multiple	15 (53.6%)	25 (75.8%)	37 (52.9%)	
Extracapsular extension of LN				**0.002**
Absence	20 (71.4%)	15 (45.6%)	51 (72.9%)	
Presence	8 (28.6%)	18 (54.4%)	19 (27.1%)	
Largest diameter of the metastatic foci in LN				**<0.001**
≤2 mm	8 (28.6%)	2 (6.1%)	25 (35.7%)	
>2 mm	20 (71.4%)	31 (93.9%)	45 (64.3%)	

MMRd: mismatch repair deficiency. p53abn: p53 abnormal. NSMP: no specific molecular profile/no surrogate marker profile. Bold-faced values indicate *p* < 0.05.

## Data Availability

All data included in this study are available upon request by contacting the corresponding author.

## Supplementary Materials

The following supporting information can be downloaded at: https://www.mdpi.com/article/10.3390/cancers18172770/s1, Figure S1. Flowchart of the patient’s enrollment; Figure S2. The representative histopathological images of extracapsular extension of lymph; Figure S3. Flowchart of the methodology for molecular classification; Figure S4. Molecular classification of endometrial carcinoma; Figure S5. Prognostic significance of the treatment selection in patients with stage IIIC endometrial carcinoma; Figure S6. Prognostic significance of the unilateral/bilateral pelvic lymph node metastasis and single/multiple lymph nodes involved in patients of stage IIIC endometrial carcinoma; Figure S7. Kaplan–Meier curves estimating PFS by invasion of cervical stroma in patients with stage IIIC endometrial carcinoma; Figure S8. Kaplan–Meier curves estimating PFS by extracapsular/intracapsular lymph node extension in the stage IIIC2 cohort; Table S1. The test proportional hazards assumptions. Univariate Cox regression analyses; Table S2. The power calculation; Table S3. Characteristics of endometrial carcinoma patients; Table S4. Clinicopathological Characteristics of Extracapsular Nodal Extension in Endometrial Carcinoma; Table S5. PFS reduction rates of clinicopathological features.
