# Supplementary material for: Extracapsular Nodal Extension as a Key Prognostic Factor in FIGO IIIC Endometrial Carcinoma: A Clinic–Pathologic and Molecular Analysis
Source: Cancers (Basel). 2026 Aug 26;18(17):2770. doi: 10.3390/cancers18172770 (PMC13564643; doi:10.3390/cancers18172770)
Supplement: Supplementary file 1 [file cancers-18-02770-s001.zip › cancers-4436558-supplementary.pdf]

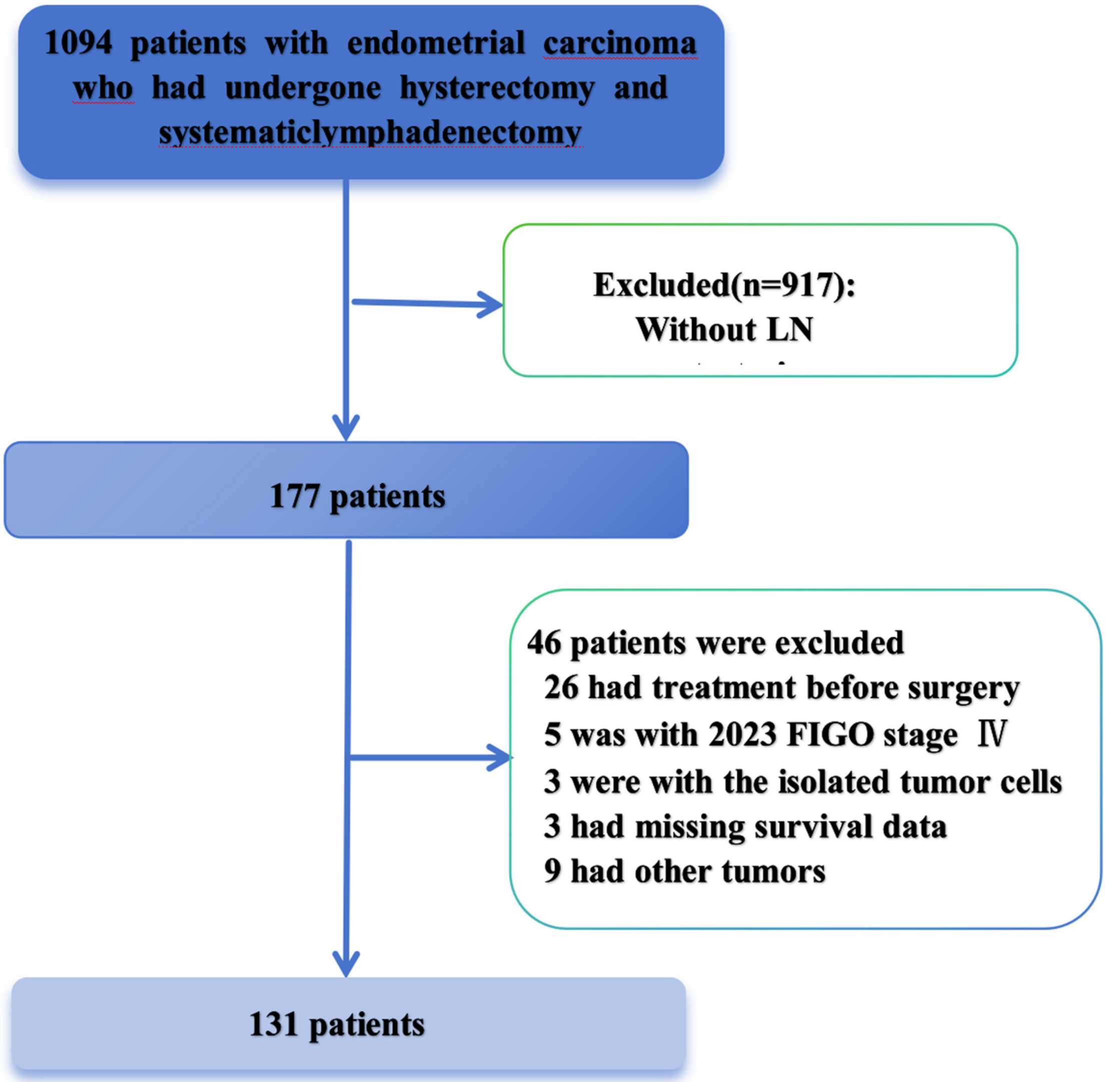

Supplementary Figure S1. Flowchart of the patient's enrollment. LN, lymph node; FIGO, International Federation of Gynecology and Obstetrics.

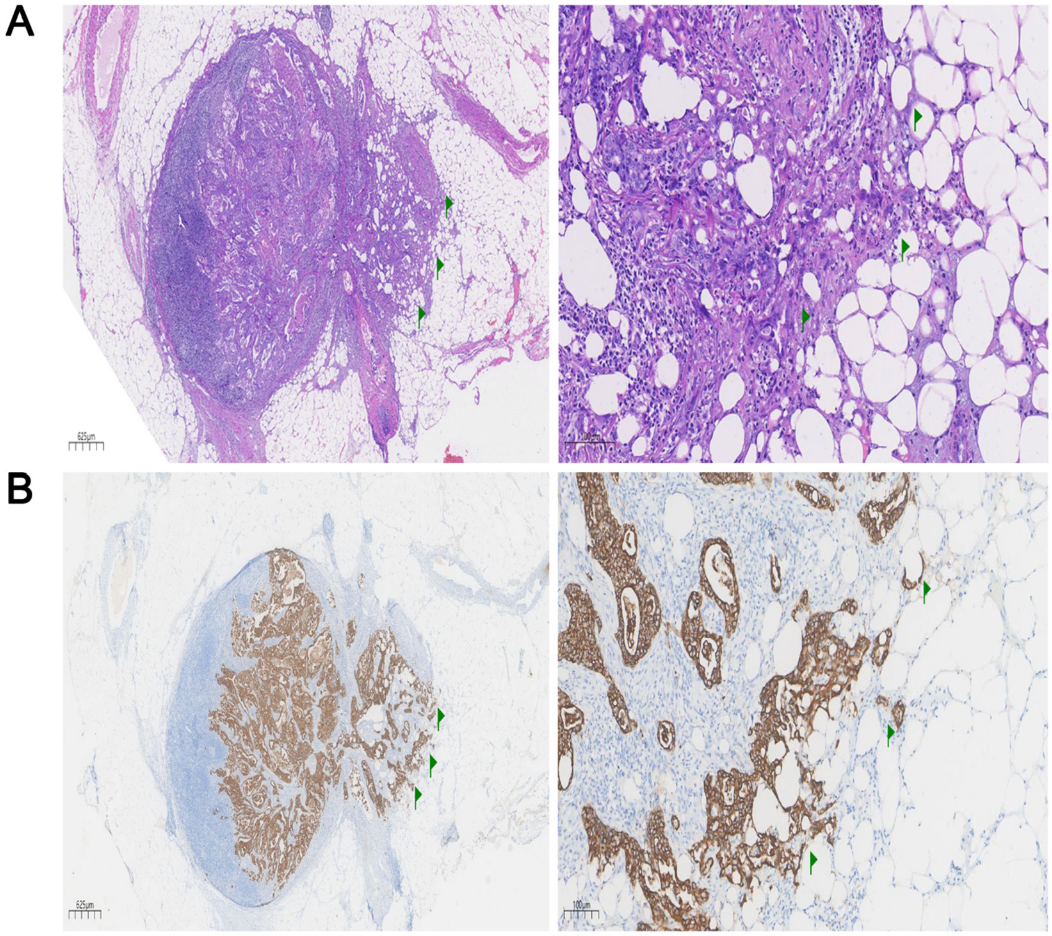

Supplementary Figure S2. The representative histopathological images of extracapsular extension of lymph.(A) Low-power (left) and high-power (right) views of hematoxylin-eosin (HE)-stained lymph node sections. (B) Corresponding low-power (left) and high-power (right) fields from pan-cytokeratin (CK-pan) immunohistochemical staining. Green flags mark regions with evident lymph node capsular disruption, where neoplastic cells extend into perinodal adipose tissue.

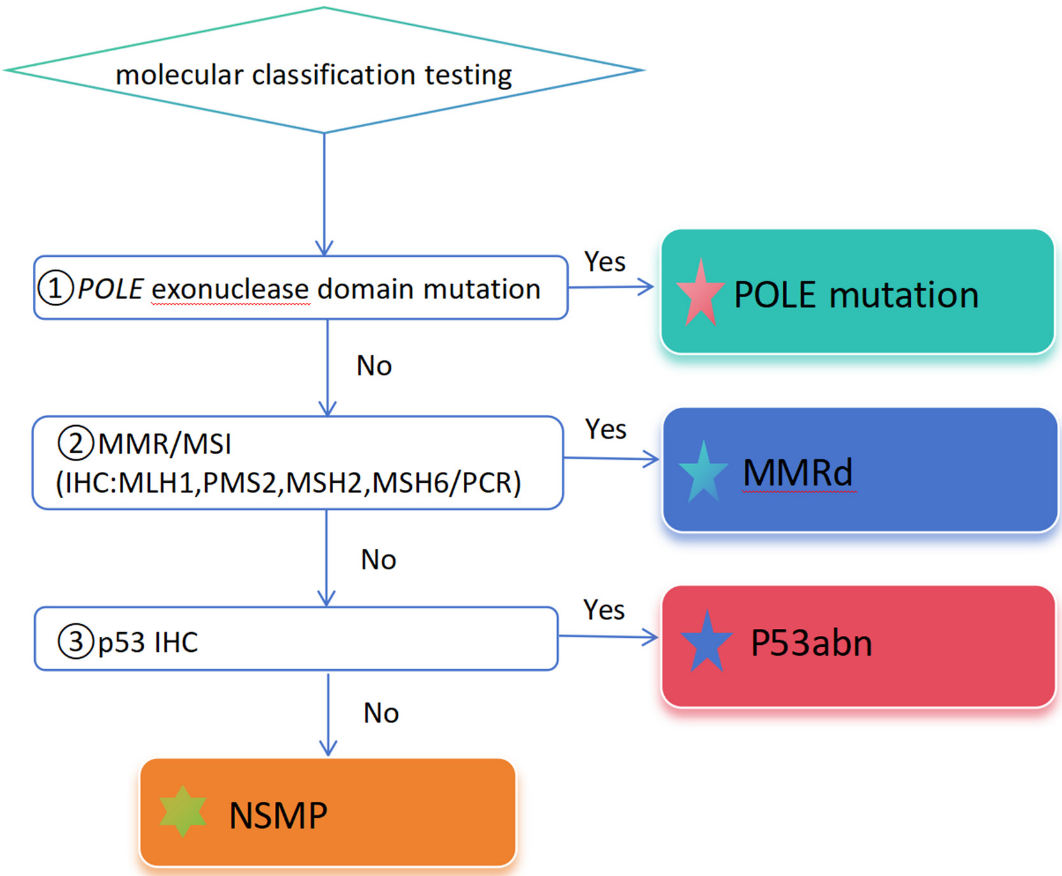

Supplementary Figure S3. Flowchart of the methodology for molecular classification. MMRd: mismatch repair deficient; MSI: microsatellite instability; IHC: immunohistochemistry; P53abn: P53 abnormal; NSMP: non-specific molecular profile.

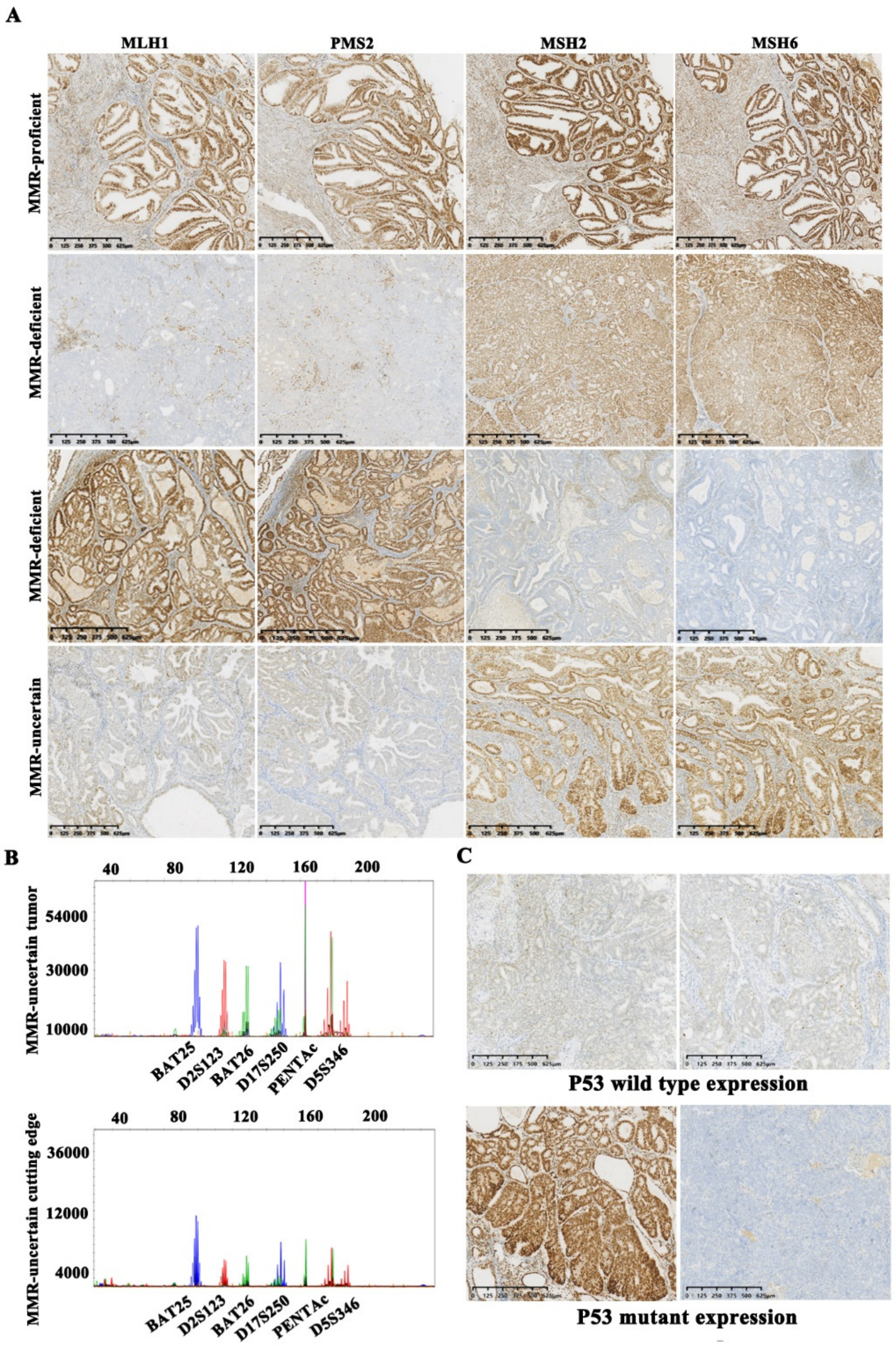

Supplementary Figure S4. Molecular classification of endometrial carcinoma. (A) Representative images of immunohistochemical staining indicative of MMR status (detected by MLH1, PMS2, MSH2, MSH6). (B) The microsatellite instability (MSI) status was assessed using fluorescence-labeled microsatellite markers (BAT25, BAT26, D2S123, D5S346, D17S250) through polymerase chain reaction (PCR). (C) Representative images of immunohistochemical staining demonstrating P53 expression.

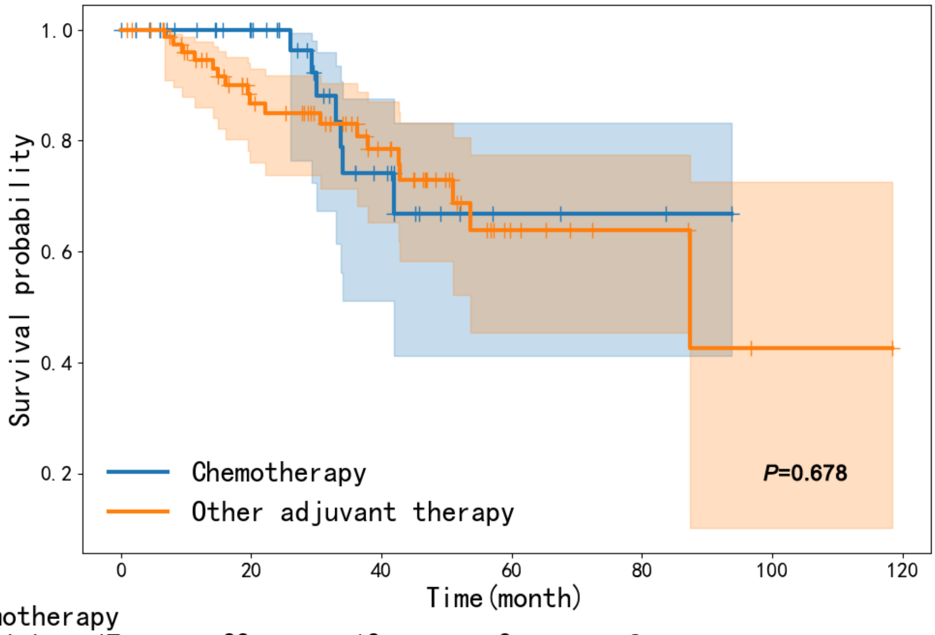

	0	20	40	60	80	100	120
**Chemotherapy**							
At risk	47	33	13	3	2	0	0
Censored	3	17	31	40	41	43	43
Events	0	0	6	7	7	7	7
**Other adjuvant therapy**							
At risk	80	52	31	8	4	1	0
Censored	0	19	36	55	59	61	62
Events	0	9	13	17	17	18	18

Supplementary Figure S5. Prognostic significance of the treatment selection in patients with stage IIIC endometrial carcinoma. Kaplan Meier curves estimating PFS by chemotherapy/other adjuvant therapy.

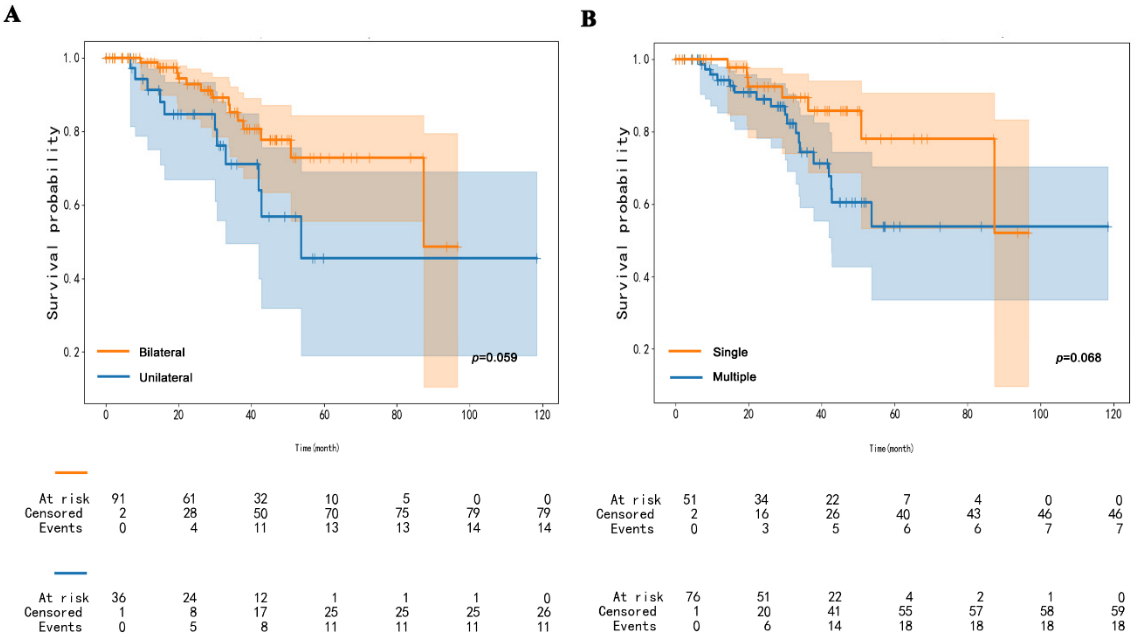

Supplementary Figure S6. Prognostic significance of the unilateral/bilateral pelvic lymph node metastasis and single/multiple lymph nodes involved in patients of stage IIIC endometrial carcinoma. (A) Kaplan Meier curves estimating PFS by unilateral/bilateral pelvic lymph node metastasis. (B) Kaplan-Meier curves estimating PFS by single/multiple lymph node involvement.

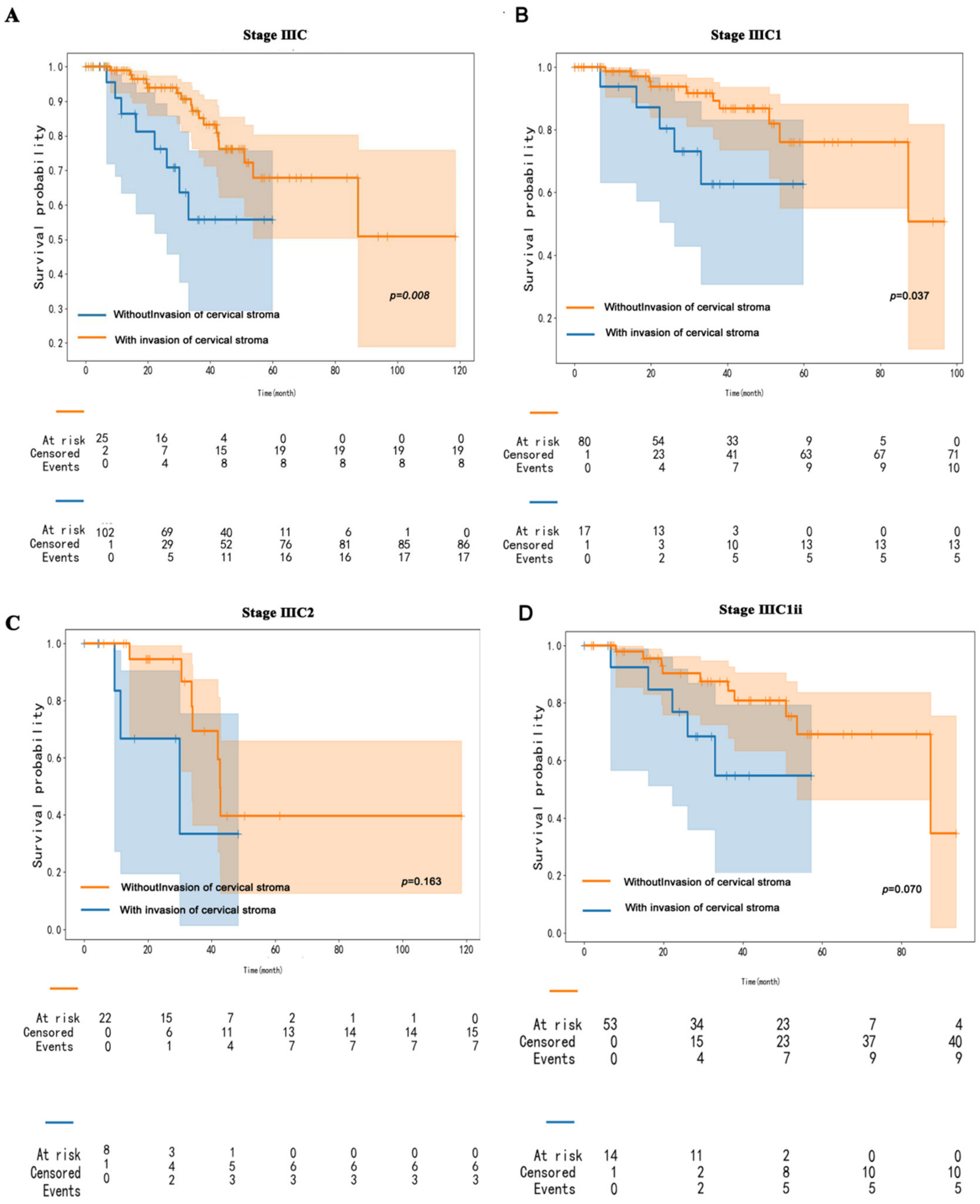

Supplementary Figure S7. Kaplan Meier curves estimating PFS by invasion of cervical stroma in patients with stage IIIC endometrial carcinoma. (A)Kaplan-Meier curves estimating PFS by cervical stroma invasion in stage IIIC patients. (B) Kaplan-Meier curves estimating PFS by cervical stroma invasion in stage IIIC1 patients. (C) Kaplan-Meier curves estimating PFS by cervical stroma invasion in stage IIIC2 patients. (D) Kaplan-Meier curves estimating PFS by cervical stroma invasion in stage IIIC1ii patients.

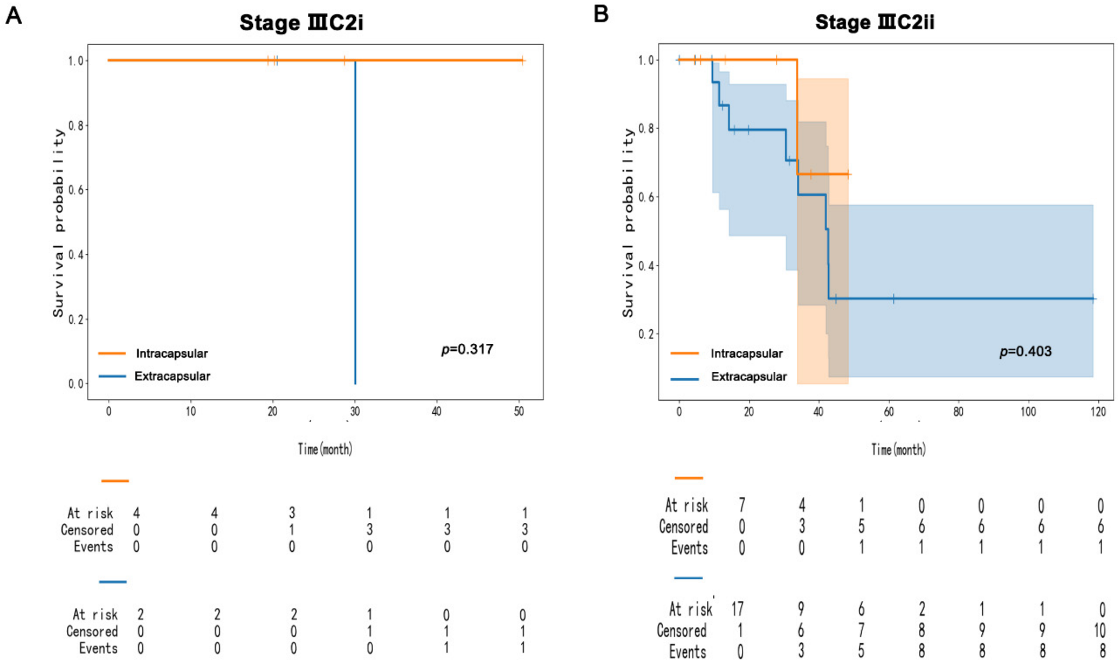

Supplementary Figure S8. Kaplan-Meier curves estimating PFS by extracapsular/intracapsular lymph node extension in the stage IIIC2 cohort.

Supplementary Table S1. The test proportional hazards assumptions.

Univariate Cox regression analyses

Variable	*P*-Value	Sperman
Location of the metastatic LNs (Pelvic and para-aortic vs Only pelvic)	0.877	0.026
Unilateral or bilateral pelvic LN metastasis	0.651	0.075
Number of involved LNs	**0.009**	0.411
Extracapsular tumor extension of LN	0.375	-0.146
Largest diameter of the metastatic foci in LN	0.278	-0.183
Lymphovascular space invasion	0.188	0.215
Histologic subtype	0.247	-0.019
Myometrial invasion	0.014	-0.392
Invasion of cervical stroma	0.108	0.262
Adnexal involvement	0.750	0.083

Multivariate Cox regression analyses

Variable	*P*-value	Sperman
Invasion of cervical stroma	0.751	-0.072
Adnexal involvement	0.148	0.319
Histologic subtype	0.296	-0.233
Extracapsular tumor extension of LN	0.852	-0.042

Supplementary Table S2. The power calculation.

Variable	Power
Largest diameter of the metastatic foci in LN	0.20
Extracapsular tumor extension of LN	**0.76**
Lymphovascular space invasion	0.25
Histologic subtype	0.61
Myometrial invasion	0.24
Invasion of cervical stroma	**0.74**
Adnexal involvement	**0.78**
Number of involved LNs	0.33
Unilateral or bilateral pelvic LN metastasis	0.39
Location of the metastatic LNs	0.49

The α level set at 0.05

Supplementary Table S3. Characteristics of endometrial carcinoma patients.

Characteristic		N (%)
Median age (range)		56.7 (33-74)
Histologic subtype		
Non-aggressive histologic types		63(48.1%)
	Endometrioid carcinoma FIGO 1	17(13.0%)
	Endometrioid carcinoma FIGO 2	46(35.1%)
Aggressive histologic types		68(51.9%)
	Endometrioid carcinoma FIGO 3	34(25.9%)
	Serous carcinoma	18(13.6%)
	Carcinosarcoma	5(3.8%)
	Mixed carcinoma	4(3.1%)
	Clear cell carcinoma	4(3.1%)
	Dedifferentiated carcinoma	1(0.8%)
	Undifferentiated carcinoma	1(0.8%)
	Squamous cell carcinoma	1(0.8%)
Myometrial invasion		
	Less than half of myometrium	49 (37.4%)
	Half or more of the myometrium	82(62.6%)
Invasion of cervical stroma		
	No	104(79.4%)
	Yes	27(20.6%)
Lymphovascular space invasion		
	No and focal	77(58.8%)
	Substantial	54 (41.2%)
Adnexal involvement		
	No	114(87.0%)
	Yes	17 (13.0%)
FIGO Stage IIIC		
	IIIC1i	46(35.1%)
	IIIC1ii	52(39.8%)
	IIIC2i	7 (5.3%)
	IIIC2ii	26(19.8%)
Adjuvant therapy		
	Chemotherapy	131(100%)
	Chemotherapy with radiotherapy	60(45.8%)
	Chemotherapy with targeted therapy	11(8.4%)
	Chemotherapy with immunotherapy	15(11.5%)

FIGO: International Federation of Gynecology and Obstetrics.

Supplementary Table S4. Clinicopathological Characteristics of Extracapsular Nodal Extension in Endometrial Carcinoma

	Without Extracapsular Nodal Extension	With Extracapsular Nodal Extension	X^2	*p* value
**Tumor size**				
**＜3cm**	19	8		
**≥3cm**	67	37	0.1242	0.725
**Adnexal involvement**				
**Absent / FIGO stage IA**	81	33		
**Others**	5	12	9.6035	**0.002**
**Cervical stromal invasion**				
**No**	70	34		
**Yes**	16	11	0.3105	0.577
**Myometrial invasion**				
**Less than half of myometrium**	38	11		
**Half or more of the myometrium**	48	34	4.1104	**0.0426**
**Lymphovascular space invasion (LVSI)**				
**Absent / focal (AE or localized)**	55	22		
**Extensive LVSI**	31	23	2.1802	0.140
**FIGO stage**				
**IIIC1i**	28	1		
**IIIC1ii**	47	24		
**IIIC2i**	4	2		
**IIIC2ii**	7	18		**0.002**
**diameter of metastatic lymph node**				
**≤0.2-2mm**	32	3		
**＞2mm**	54	42	12.5585	**0.0003**
**Number of metastatic lymph nodes**				
**Single metastatic node**	44	10		
**＞1 metastatic nodes**	42	35	9.0525	**0.003**
**Laterality of lymph node metastasis**				
**Unilateral**	70	24		
**Bilateral**	16	21	10.1358	**0.001**
**Pattern of lymph node metastasis**				
**Pelvic-only nodal metastasis**	75	25		
**Concurrent pelvic and para-aortic nodal metastasis**	11	20	9.7341	**0.002**
**Para-aortic lymph node status**				
**No**	34	12		
**Yes**	11	20		
**not dissected**	41	13	9.7314	**0.002**

Clinicopathological Characteristics of Extracapsular Nodal Extension in Endometrial Carcinoma

	Without Extracapsular Nodal Extension	With Extracapsular Nodal Extension	X^2	*p* value
**Molecular classification**				
**NSMP**	51	19		
**POLE**				
**dMMR**	20	8		
**P53abn**	15	18	1.802	0.406
**Histologic subtype**				
**Non-aggressive histologic types**	55	8		
**Aggressive histologic types**	31	37	23.4167	**0.000**

Supplementary Table S5.PFS reduction rates of clinicopathological features

	3-year mean percentage reduction in PFS (%)
**Histologic subtype (Aggressive vs Non-aggressive)**	**0.6**
**Molecular classification**	**6.2<br>27.5**
**Nodal metastatic pattern (Concurrent pelvic and para-aortic metastasis vs Pelvic-only metastasis)**	**15.5**
**Laterality of lymph node metastasis (Bilateral vs Unilateral)**	**7.1**
**Number of metastatic lymph nodes (Multiple vs Single)**	**1.8**
**Extracapsular nodal extension (Present vs Absent)**	**22.0**
**diameter of metastatic lymph node (Macrometastasis vs Micrometastasis)**	**6.3**
**Lymphovascular space invasion (LVSI) (Extensive vs Absent)**	**-11.7**
**Myometrial invasion (>1/2myometrium vs 1/2 myometrium)**	**5.9**
**Cervical stromal invasion (Present vs Absent)**	**50.7**
**Adnexal involvement (Present vs Absent)**	**53.5**
**Tumor size (≥ 3 cm vs < 3 cm)**	**9.7**
